# Single-cell long-read sequencing in human cerebral organoids uncovers cell-type-specific and autism-associated exons

**DOI:** 10.1016/j.celrep.2023.113335

**Published:** 2023-10-26

**Authors:** Yalan Yang, Runwei Yang, Bowei Kang, Sheng Qian, Xin He, Xiaochang Zhang

**Affiliations:** 1 Department of Human Genetics, Neuroscience Institute, The University of Chicago, Chicago, IL 60637, USA; 2 These authors contributed equally; 3 Lead contact

## Abstract

Dysregulation of alternative splicing has been repeatedly associated with neurodevelopmental disorders, but the extent of cell-type-specific splicing in human neural development remains largely uncharted. Here, single-cell long-read sequencing in induced pluripotent stem cell (iPSC)-derived cerebral organoids identifies over 31,000 uncatalogued isoforms and 4,531 cell-type-specific splicing events. Long reads uncover coordinated splicing and cell-type-specific intron retention events, which are challenging to study with short reads. Retained neuronal introns are enriched in RNA splicing regulators, showing shorter lengths, higher GC contents, and weaker 5′ splice sites. We use this dataset to explore the biological processes underlying neurological disorders, focusing on autism. In comparison with prior transcriptomic data, we find that the splicing program in autistic brains is closer to the progenitor state than differentiated neurons. Furthermore, cell-type-specific exons harbor significantly more *de novo* mutations in autism probands than in siblings. Overall, these results highlight the importance of cell-type-specific splicing in autism and neuronal gene regulation.

## In brief

Yang et al. perform single-cell long-read sequencing in the human cerebral organoids, identifying thousands of uncatalogued isoforms, differentially spliced exons, and retained introns. Their results reveal the enrichement of autism-associated exons and *de novo* mutations in cell-type-specific exons.

## Graphical Abstract

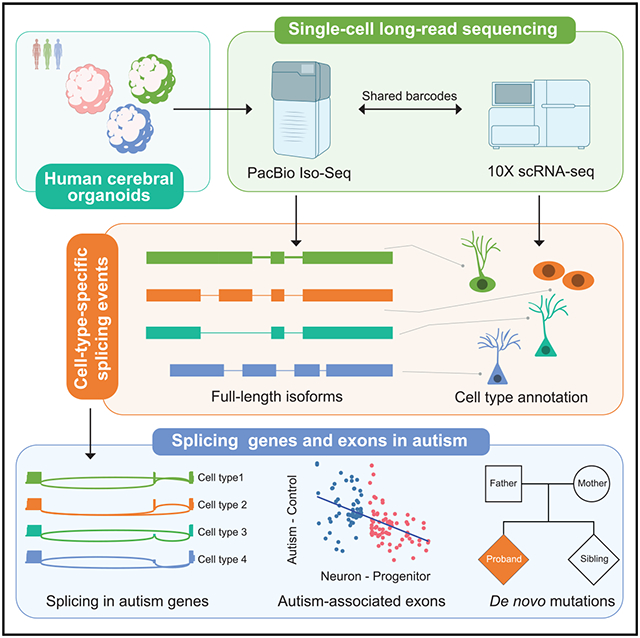

## INTRODUCTION

Alternative splicing (AS) regulates over 90% of human genes, and the human brain displays the most distinctive AS pattern compared to other tissues.^1-3^ AS has been recognized as a major mechanism for generating protein diversity,^4,5^ and dysregulation of AS has been linked to the pathogenesis of neurological disorders.^6^ Deep intronic mutations disrupting alternative exons have been reported to cause human brain malformation and epilepsy.^7,8^ These findings indicate the critical roles of AS in human neural development and disease etiology.

Autism spectrum disorder (ASD) impairs social interaction and affects about 2.8% of 8-year-old children in the United States (CDC, https://www.cdc.gov/media/releases/2023/p0323-autism.html). Mutations in AS regulators such as RBFOX1 have been shown to cause ASD and intellectual disability.^9^ Comparisons of postmortem brains from patients with ASD and healthy donors uncovered dysregulated AS networks controlled by RBFOX1, nSR100, or PTBP2 proteins.^10-13^ On the other hand, ASD genes have been reported to express different isoforms that are important for neural development and synaptic connectivity.^14^

Single-cell RNA sequencing (scRNA-seq) uncovers unprecedented cell-type heterogeneity in human tissues,^15^ but the extent of cell-type-specific pre-mRNA splicing remains largely uncharted. Current scRNA-seq platforms are predominantly built on read counts at the 3′ or 5′ end of polyadenylated transcripts and do not generate sufficient coverage for splice junctions.^16,17^ On the other hand, short-read single-cell full transcriptome analysis is cost prohibitive, has relatively lower throughput, and has only been done in limited mouse brain studies.^18,19^ Recent single-cell short-read surveys of the adult mouse brain uncovered splicing diversity across brain cell types.^20,21^ It remains unclear how human genes are differentially spliced between cell types in neural development. Short-read scRNA-seq also misses the opportunity to uncover coordinated splicing events. To overcome these challenges, we have integrated droplet-based scRNA-seq with long-read sequencing to profile cell-type-specific splice isoforms, a comparable approach to previous reports in postnatal brains.^22^

The developing human brain has been increasingly modeled by cerebral organoids for developmental mechanisms and human diseases such as autism.^23-27^ While the cell-type compositions of fetal human brains and cerebral organoids have been well studied and compared,^28^ the expression of full-length splice isoforms in human neural development remains undescribed. Here, we seek to fill this gap by single-cell long-read sequencing in human cerebral organoids. This study identifies previously unannotated exons and splice isoforms, uncovers pervasive splicing changes across neural cell types, and suggests that autism-associated exons and *de novo* mutations are enriched in cell-type-specific exons.

## RESULTS

### Single-cell and long-read RNA sequencing of human cerebral organoids

**Figure 1.**
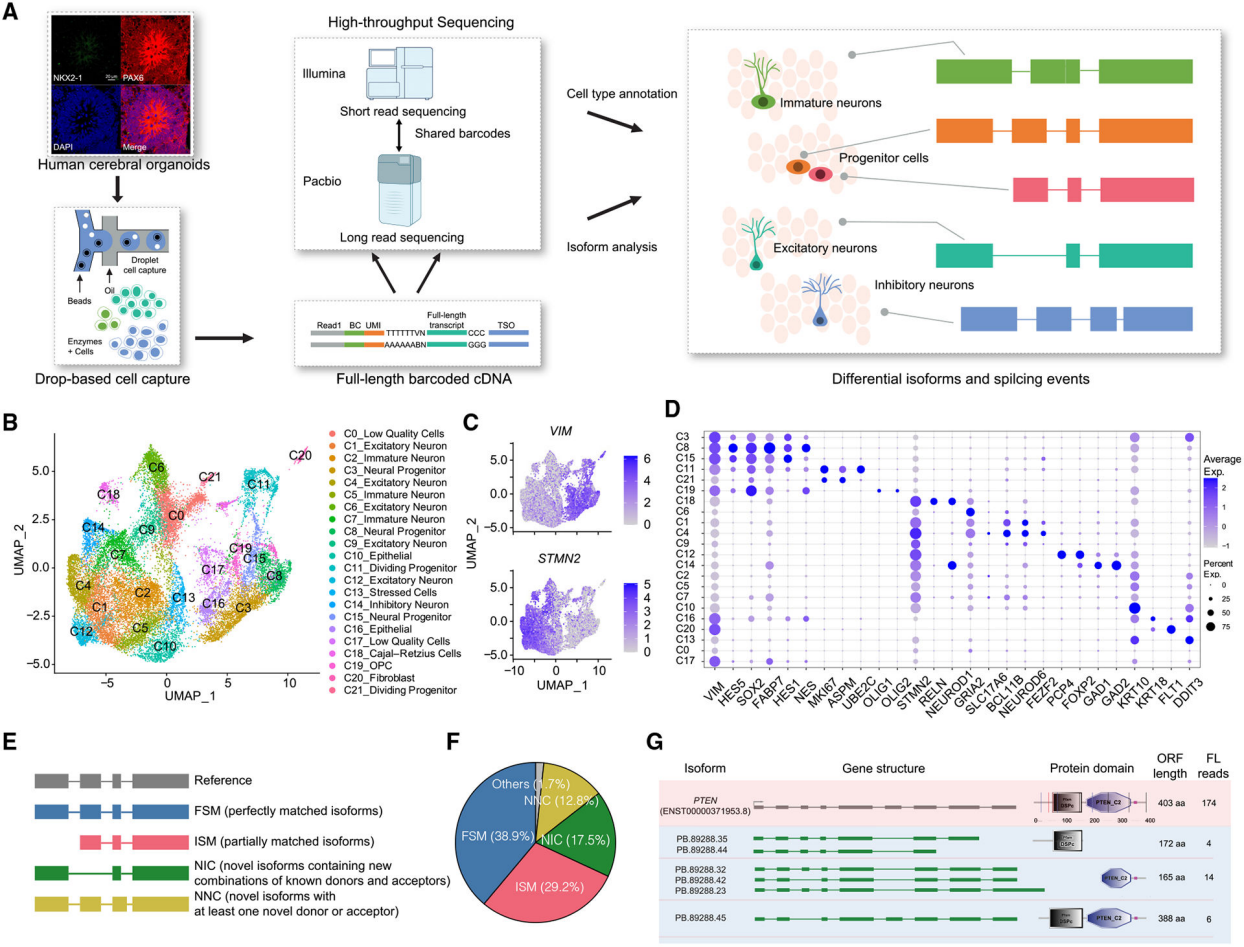
scIso-seq in human cerebral organoids uncovers previously unannotated splice isoforms (A) The workflow of scIso-Seq: integrating scRNA-seq and Iso-seq to identify cell-type-specific splicing events. (B) UMAP visualization of scRNA-seq data from human cerebral organoids. Cells are color-coded by annotated cell types. (C) Feature plots showing the expression of neuronal marker *STMN2* and neural progenitor marker *VIM*. (D) Representative marker genes in each cell type. Average expression levels were derived from logarithm-scaled normalized counts based on the scRNA-seq data. The size of the dot indicates the percentage of cells expressing each gene. (E) Types and illustrations of identified isoforms by Iso-seq. (F) A pie chart showing the ratio of different types of isoforms. NIC (17.5%, 17,985) and NNC (12.8%, 13,196) represent previously annotated isoforms. (G) Exon-intron structures and predicted protein domains (SMART) of previously unannotated splice isoforms identified in the *PTEN* gene. PTEN consists of a catalytic N-terminal phosphatase domain and a C-terminal C2 domain. The numbers of full-length reads that support these isoforms are shown.

We performed single-cell long-read sequencing (single-cell isoform sequencing [scIso-seq]) to investigate cell-type-specific splicing in human cerebral organoids (Figure 1A). Single-cell full-length (FL) cDNAs produced from the organoids were barcoded by the 10× Genomics platform to enable cell-of-origin identification and then split into two pools: one pool was tagmented and used for high-throughput scRNA-seq to identify cell types and measure gene expression. The other pool was used for PacBio FL Iso-seq to identify FL isoforms. We assigned cell-type information to long reads using shared cell barcodes to identify cell-type-specific splice isoforms.

To capture neural progenitors and neurons, we generated dorsal forebrain organoids from human induced pluripotent stem cells (iPSCs) based on a published protocol.^29,30^ Briefly, three independent iPSC lines (19101 and 28126, male; 21792, female) were cultured in an AggreWell-800 plate for 24 h to form three-dimensional spheroids with a diameter of about 170 μm (Figures S1A and S1B). Neutralization of spheroids was guided by two SMAD pathway inhibitors (dorsomorphin and SB-431542), followed by treatment with epidermal growth factor (EGF) and fibroblast growth factor 2 (FGF2) to allow neurogenesis and then brain-derived neurotrophic factor (BDNF) and neurotrophin 3 (NT3) to promote maturation (Figure S1C). After 1-month differentiation, brain organoids showed strong expression of a dorsal forebrain marker PAX6, with little expression of a ventral marker NKX2-1 (Figure S1C). TBR1-positive or RELN-positive neurons appeared on day 37, while CTIP2-positive neurons were not detected on the same day (Figures S1D and S1E). SOX2-positive neural progenitors largely aggregated in rosette-like structures, and neurons with strong expressions of TBR1, CTIP2, and RELN were distributed at the periphery of rosette-like structures in 1.5-month-old organoids (Figures S1F and S1G). SATB2-positive neurons showed up in organoids at later stages (e.g., day 106; Figure S1H). The order of emergence for TBR1-, CTIP2-, and SATB2-positive neurons in cerebral organoids recapitulated that in human brain development.^31^

scRNA-seq was performed on six batches of cerebral organoids at 3–5 months of growth (Figures S1I and S1J). After filtering out low-quality cells (over 5,000 or below 300 detected genes, or over 5% mitochondrial gene counts), a total of 28,290 cells were projected into an adjusted two-dimensional space by uniform manifold approximation and projection (UMAP). All cells were classified into 22 clusters with Seurat and annotated based on marker genes (Figures 1B-1D and S1I). The neurons expressing *STMN2* included five clusters (C1, C4, C6, C9, and C12) of excitatory neurons expressing *GRIA2, SLC17A6, BCL11B,* and *NEUROD6*; one cluster (C14) of inhibitory neurons expressing *GAD1* and *GAD2*; and one cluster (C18) of Cajal-Retzius cells expressing *RELN.* The C19 cluster expressing *OLIG1* and *OLIG2* was annotated as oligodendrocyte progenitor cells (OPCs). The progenitors marked by *VIM* expression included three clusters (C3, C8, and C15) of neural progenitors expressing *HES5, SOX2,* and *NES* and two clusters (C11 and C21) of dividing progenitors expressing *MKI67, APSM,* and *UBE2C* (Figures 1C, 1D, and S1K).

Three of the six organoid samples showing balanced cell types and cell numbers were selected for Iso-seq (19101_D137 [day 137], 28126_D84, and 21792_D145). Each of the organoids was sequenced on two or three SMRT cells (PacBio Sequel II), yielding over 26.0 million circular consensus sequence (CCS) reads with a median length of over 1,000 bp (Figure S2A; Table S1). After processing the raw reads and annotating the transcripts, we obtained 12.47 million FL non-chimeric (FLNC) reads and identified a total of 103,007 non-redundant isoforms that were mapped to 16,597 genes, with 70.8% of genes assigned to more than one isoform (Figure S2B; Table S2). Compared with the known isoforms in GENCODE (v.40), the length of detected isoforms was significantly longer (median 1,514 bp vs. 1,396 bp, Figure S2C), suggesting that the long-read sequencing captured FL transcripts.

We classified detected isoforms into four major groups based on the GENCODE v.40 human reference transcriptome (Figure 1E). Among the 103,007 detected isoforms, 38.9% (40,057) were characterized as full-splice matches (FSMs) to existing annotations in GENCODE and 29.2% (30,056) were incomplete-splice matches (ISMs; corresponding to isoforms that partially match to existing annotations). Importantly, 17.5% (17,985) of all the isoforms were previously unannotated in catalog (NIC; corresponding to isoforms containing unannotated combinations of known donors and acceptors) and 12.8% (13,196) were unannotated not in catalog (NNC; corresponding to isoforms with at least one unannotated donor or acceptor). Besides, we identified a proportion of isoforms (others, 1.7%) classified as antisense (n = 231), genic (n = 158; isoforms that overlap with intron), intergenic (n = 384; isoform in intergenic regions), and fusion (n = 940; transcriptional readthrough between two or more adjacent genes; Figure 1F). Characteristics analysis of the unannotated isoforms (NNC and NIC) suggested that they exhibited comparable quality to known FSM isoforms (Figures S2D and S2E). We performed pairwise comparisons at the exon and isoform levels and found a high correlation between the three long-read samples (Figures S2F-S2H), suggesting the splice isoforms are reproducible between organoid samples derived from independent iPSC lines.

The FL transcriptome data greatly expanded the isoform catalog for cell types in the human cerebral organoids. For example, we identified 13 FSM, 8 NIC, and 8 ISM isoforms in the *PTEN* gene, which is a tumor suppressor and one of the most extensively studied autism genes.^32^ Analyses of predicted protein sequences suggest that the previously unannotated *PTEN* splice forms may lead to the deletion of the catalytic N-terminal phosphatase domain or the shortening of the C2 domain (Figure 1G). Moreover, we detected 4,820 exons that are not annotated in GENCODE v.40 and RefSeq (Table S3). Over three-quarters of them (n = 3,628, 75.3%) were found in the known genes, including 111 previously unannotated exons in SFARI Autism Genes, such as the exon (chr5:138,380,173–138,380,264) in the *KDM3B* gene (SFARI gene score = 1; Figure S2I). Meanwhile, we found that 9.9% (n = 3,092) of unannotated isoforms and 8.8% of unannotated exons were validated in published long-read data of the human cerebral cortex^33^—while our organoid samples were sequenced at a much higher read depth, these results suggest that the scIso-seq uncovers previously unannotated splice isoforms that are biologically relevant.

Compared with short-read sequencing, long-read sequencing makes it feasible to investigate coordinated splicing events. In total, we identified 4,137 significantly co-regulated exon pairs (∣log-odds ratio∣ ≥ 1 and false discovery rate [FDR] < 0.001) in 541 genes, including 3,975 mutually inclusive exons and 162 mutually exclusive exons (MXEs) (Table S3). For example, we detected the documented MXEs in *MACROH2A1* and *TPM3* and the mutually inclusive exons in *ACTR10* and *TBRG4* (Figures S2J and S2K). Overall, the scIso-seq of the human cerebral organoids uncovered 4,820 previously unannotated exons, 4,137 coordinated splicing events, and 31,181 previously unannotated splice isoforms.

### scIso-seq uncovers extensive alternative exon usage in human cerebral organoids

**Figure 2.**
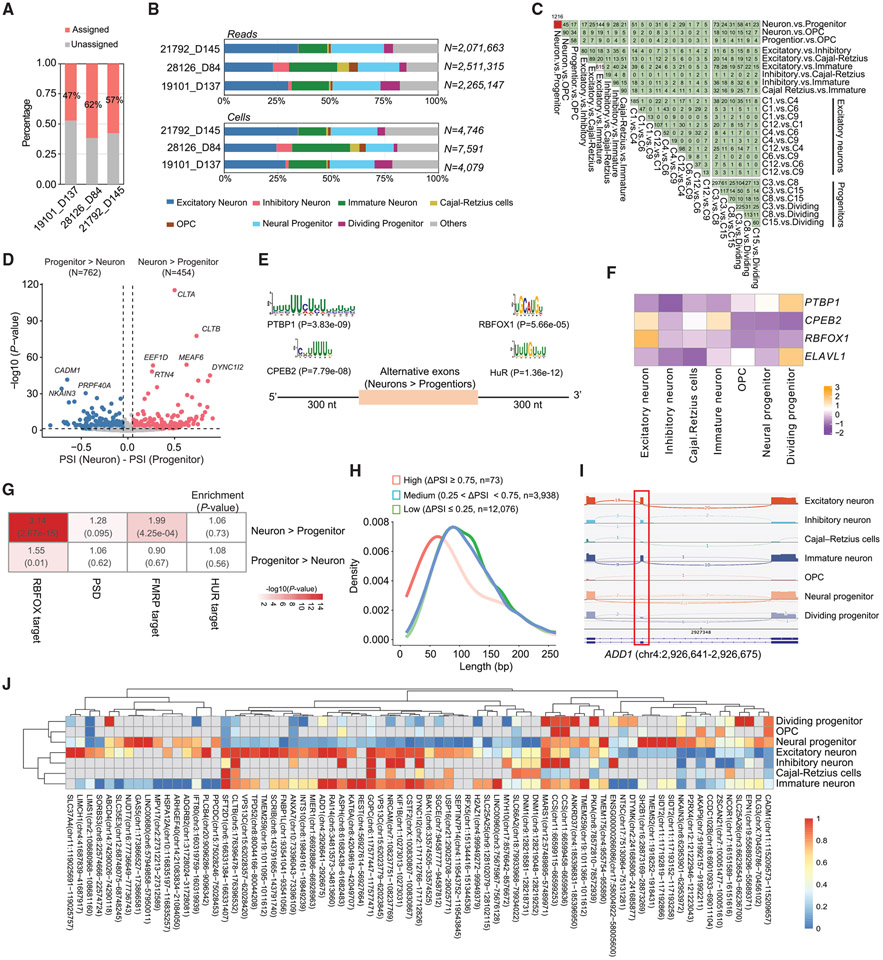
Cell-type-specific exons in human cerebral organoids (A) A bar plot showing the ratios of long reads that were assigned to specific cell types. Cell types were annotated based on scRNA-seq (Figure 1A). (B) The distribution of Iso-seq reads and cells across seven major cell types. (C) Pairwise comparisons showing the distributions of DSEs detected between different cell types at three hierarchical levels. (D) A volcano plot showing DSEs between neurons and neural progenitors. (E) Sequence motifs enriched in the flanking introns of neuron-specific DSEs. The motifs were annotated by XSTREME. (F) A heatmap based on the scRNA-seq data showing higher expression of *RBFOX1* and *CPEB2* in neurons and higher expression of *PTBP1* and *ELAVL1* in dividing progenitors. (G) Enrichment analysis of genes with DSEs between neurons and progenitors with genes encoding PSD proteins and targets of FMRP, HuR, and RBFOX1. The odds ratios and p values were calculated based on Fisher’s exact tests. (H) Density plot of the exon variability across the seven major cell types, with exon length showing on the x axis. Colors indicate the discrete categories of variability. (I) Sashimi plots from scIso-seq showing a neuron-specific exon in the *ADD1* gene. (J) Heatmap showing the PSI values of the highly variable exons (n = 73) across cell types.

We retrieved cell barcodes for the 12.47 million FLNC reads and found that 54.9% of these reads could be assigned to annotated cell types (Figure 2A). Comparable to the distribution of cells and reads in scRNA-seq, the Iso-Seq reads were assigned to seven major cell types such as excitatory neurons, immature neurons, and neural progenitor cells (Figure 2B). We further analyzed differential splicing events such as skipped exons (SEs), alternative 5′ splice sites (A5SSs), and alternative 3′ splice sites (A3SSs) between cell types at three levels using FLAIR^34^ and identified (1) differential AS events among neurons, progenitors, and OPCs; (2) differential AS events between neuronal types; and (3) differential AS events between subtypes of excitatory neurons and between subtypes of progenitors (Figures 2C and S3A-S3E). A total of 2,393 differentially spliced exons (DSEs; ∣ΔPSI (percent spliced in) ∣ ≥ 5% and adjusted p [adj.p] < 0.05) were identified from 25 pairwise comparisons, including 1,216 DSEs between neurons and progenitors (Figures 2C and 2D; Table S4). Most of the genes (1,486/1,634 = 90.9%) that contained DSEs were also differentially expressed at the isoform level (Figure S3F).

The most significant DSEs between neurons and progenitors included known and unannotated alternatively spliced exons, such as SEs in *CLTA, CLTB, DYNC1I2,* and *MEAF6* (Figure 2D). Genes with DSEs were significantly enriched in biological processes for RNA splicing, brain development, and cellular localization and transport (Figures S3G and S3H). Genes harboring neuron-specific exons were enriched for postsynaptic density (PSD) proteins^35^ (Figure 2G). For the 165/1,216 neuron-progenitor DSEs that were also reported in a previous microexon study,^11^ 97.0% of them (160 exons) showed consistent directions of splice change between neural and non-neural tissues (Figure S3I), confirming the reliability of our scIso-seq DSE pipeline.

We used XSTREME^36^ to identify cell-type-specific *cis*-regulatory sequence motifs enriched in the flanking introns of DSEs. For the DSEs that showed higher inclusion in the neurons, the binding motifs of PTBP1 and CPEB2 were enriched in the upstream introns, and the binding motifs of RBFOX1 and HuR/ELAVL1 were enriched in the downstream introns (Figure 2E). Consistent with this, *RBFOX1* and *CPEB2* showed high expression in neurons, while *PTBP1* and *ELAVL1* were enriched in progenitors (Figures 2F and S3J). Meanwhile, genes with exons that showed higher inclusion in neurons were also enriched in the targets of RBFOX1^37^ and FMRP^38^ but not in HUR targets^39^ (Figure 2G).

Our comparison strategy identified cell-type-defining DSEs. For example, there were 615 DSEs between excitatory neurons and immature neurons and eighty DSEs between excitatory neurons and inhibitory neurons (Figure 2C), which were significantly enriched in Gene Ontology (GO) terms of brain development and negative regulation of axon extension. Using alternative exons that were detected in the seven major cell types (≥ 10 reads in two or more cell types), we calculated their PSI values and considered the maximal ΔPSI between these cell types. We found that the most variable exons (max∣ΔPSI∣ ≥ 0.75) had significantly shorter lengths (Figure 2H). We identified 73 highly variable switch-like exons, such as exon 15 in *ADD1,* which were included in neurons but skipped in progenitors (Figure 2I).^40^ Among the 68 genes with highly variable exons, nine (*DYNC1I2, NRCAM, KIF1B, ADD1*, *DNM1, DLG3, VPS13C, KAT6A,* and *DTYMK*) were identified as causal for neurodevelopmental disorders, as indicated in OMIM.^41^ Clustering analysis based on the PSI values of these exons distinguished neurons from progenitors (Figure 2J). These results suggest that cell-type-specific exons are prevalent in neural development.

### Differentially retained introns between neural cell types

**Figure 3.**
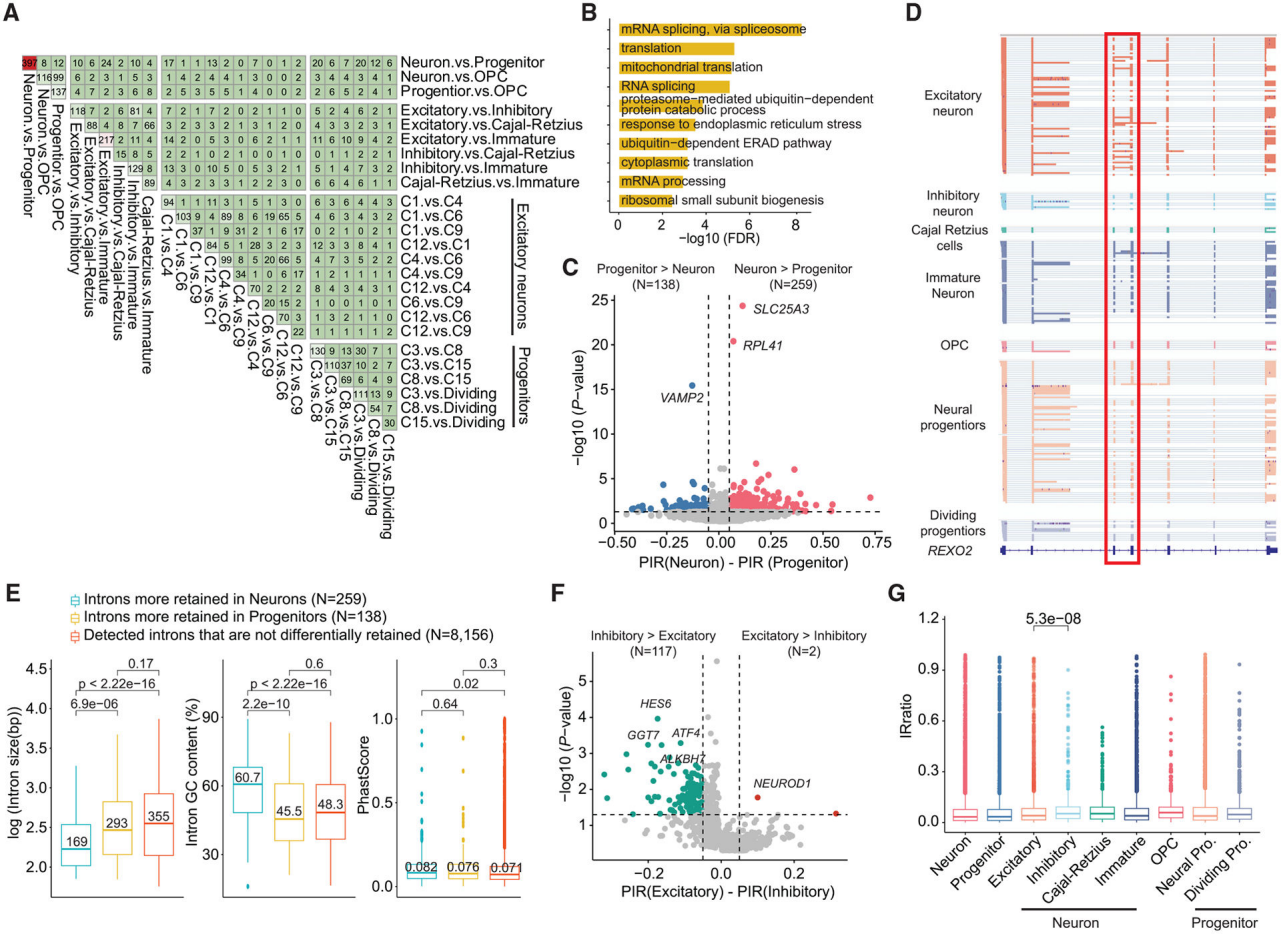
Differentially retained introns in human cerebral organoids (A) Pairwise comparisons showing cell-type-specific DRIs detected between different cell types at three levels. (B) Gene Ontology analysis of the genes with DRIs. (C) A volcano plot showing introns that are differentially retained between neurons and neural progenitors. PIR, percent intron retention. (D) IGV browser tracks showing that *REXO2* intron 3 was retained in excitatory neurons. (E) Boxplots showing the length, GC content, and PhastCons conservation score, respectively, of introns that are more retained in neurons, introns that are more retained in progenitors, and introns that are not differentially retained between neurons and progenitors. Mann-Whitney U test was used for statistical analysis. (F) A volcano plot showing introns that are differentially retained between excitatory and inhibitory neurons. (G) Boxplots showing the IR ratio of retained introns in different cell types. Mann-Whitney U test was used for statistical analysis.

Intron retention (IR) profoundly affects gene expression by retaining transcripts in the nucleus or by promoting degradation.^42^ Previous studies suggested that neural cell types exhibited a higher proportion of retained introns when compared with other tissues.^43^ However, it has been challenging to quantify introns by short-read sequencing.^44^ Using the long-read sequencing data of human cerebral organoids, we identified IRs by IRFinder-S^45^ and compared IRs from different cell types. A total of 1,427 differentially retained introns (DRIs) were identified (∣ΔPIR∣ ≥ 5% and FDR < 0.05; Figure 3A; Table S4). The genes with DRIs were enriched in GO terms related to RNA splicing and translation (Figure 3B). KEGG analysis suggested that these genes were enriched in pathways of the spliceosome, the ribosome, amyotrophic lateral sclerosis (ALS), Huntington’s disease, and Parkinson’s disease (Figure S4A).

There are 397 DRIs between neurons and progenitors. IRs are more prevalent in neurons (n = 259) than in progenitors (n = 138) (Figure 3C). A DRI (intron 3) in the *REXO2* gene was partially retained in neurons while spliced out in progenitors (Figure 3D). Further analysis suggested that this IR almost exclusively originated from excitatory neurons, with the upstream and downstream exons of this intron mutually associated (Figure 3D). We then investigated whether the significantly elevated introns in neurons possessed unique features. We found that the introns that showed higher retention in neurons tend to have shorter lengths, higher GC content, higher conservation scores, and relatively weaker 5′ splice sites but no significant difference in the strength of the 3′ splice sites (Figures 3E and S4B). These results suggest that the weaker 5′ splice site and the higher GC content may play a role in IR in neurons.

Interestingly, the number of DRIs between excitatory neurons and inhibitory neurons (n = 119) was much larger than comparisons between subtypes of neuron, and 117/119 excitatory-inhibitory neuron DRIs showed higher retention in inhibitory neurons (Figure 3F), though excitatory neurons contained more reads and cells than those in inhibitory neurons (Figure 2B). Using a published RNA-seq dataset of mouse excitatory and inhibitory neurons,^46^ we found that more introns were retained in mouse vasoactive intestinal peptide (VIP) inhibitory neurons than in excitatory neurons (Figure S4C). We further calculated the average IR ratio in each cell type and confirmed that the retained introns in inhibitory neurons showed significantly higher inclusion than those in excitatory neurons (Figure 3G). These results indicate that a group of introns are differentially retained between neuronal cell types.

### DSEs inform the splicing disruption and genetics of autism

**Figure 4.**
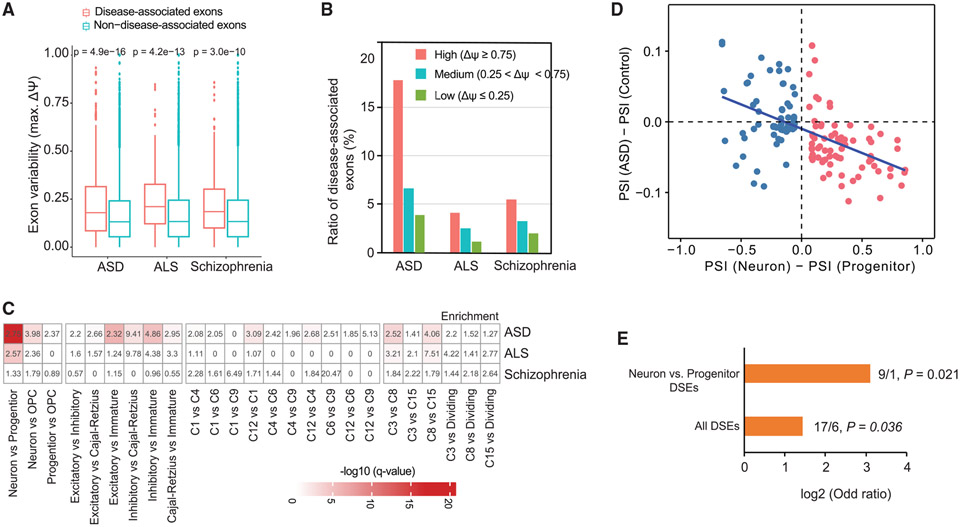
DSEs are associated with ASD etiology (A) Boxplots showing exon variability for disease-associated exons compared to alternative exons with no known association with that disease. p values were obtained from a two-sided Wilcoxon rank-sum test. (B) The ratio of disease-associated exons in different variability groups (high, medium, and low). (C) Enrichment analysis of DSEs between cell types with alternative exons identified in three different neurological disorders. The odds ratios and p values were calculated based on Fisher’s exact tests. (D) ΔPSI values of neuron-progenitor DSEs are negatively correlated with ΔPSI values of DSEs between individuals with ASD and control subjects. The correlation was calculated by Pearson’s correlation coefficient. (E) Autism probands carry significantly more DNMs in DSEs. The x axis is the odds ratio. On the top of each bar, we labeled the number of DNMs in ASD probands and siblings and the p values based on Fisher’s exact tests.

Dysregulation of AS has been associated with the pathogenesis of neurological diseases, and we investigated the inclusion variability across cell types for exons associated with ASD,^12^ ALS,^47^ and schizophrenia.^48^ These disease-associated exons were defined based on differential splicing between postmortem brain samples from affected individuals and controls. We found that disease-associated exons showed significantly higher variability across cell types than non-disease-associated exons, with the most significant difference observed in ASD (Figures 4A and 4B). Furthermore, DSEs between neurons and progenitors exhibited significant enrichment in ASD-associated exons but not in ALS- or schizophrenia-associated exons (Figure 4C). In addition, the significant enrichment of disease-associated exons was observed for comparisons between certain excitatory neuron subtypes, progenitor subtypes, and neuron classes (Figure 4C). Exons skipped in individuals with ASD tend to have higher inclusion in neurons than in progenitors, whereas exons included in individuals with ASD tend to have higher inclusion in progenitors (Figure S5A). Among the 144 exons that were differentially spliced between neurons and progenitors, and between individuals with ASD and control subjects, we found that their splicing changes (ΔPSIs) are negatively correlated between these two comparisons (p = 6.4e–11; Figure 4D). These observations suggest that the AS pattern observed in individuals with ASD appears closer to the splicing state in progenitors than in differentiated neurons.

We studied the pattern of *de novo* mutations (DNMs) in ASD as another way of exploring the connection between splicing programs and ASD. Previous studies have suggested that DNMs predicted to affect splicing are significantly enriched in ASD probands.^49^ Using the DNMs identified in whole-genome sequencing data,^50^ we confirmed that splicing mutations (SpliceAI score > 0.2) around exon junction regions (400 bp upstream and downstream) were enriched in ASD probands (Fisher’s exact test, odds ratio [OR] = 1.51, p = 0.007). This analysis included all exons in the genome. We hypothesize that DSEs between different cell types are particularly important for the genetics of ASD. We thus tested the enrichment of exonic DNMs in DSEs. We found that DSEs showed a significantly higher enrichment of DNMs in ASD probands compared to their siblings (log2[OR] = 1.43, Fisher’s exact test p = 0.036). Notably, when we specifically focused on DSEs between neurons and progenitors, the enrichment was even more pronounced. Among these DSEs, we identified 9 DNMs in ASD probands, whereas only 1 DNM was found in DSEs in their siblings (log2[OR] = 3.10, Fisher’s exact test p = 0.021, Figure 4E). These results suggest that the disruption of DSEs may contribute to ASD.

### AS of ASD risk genes

**Figure 5.**
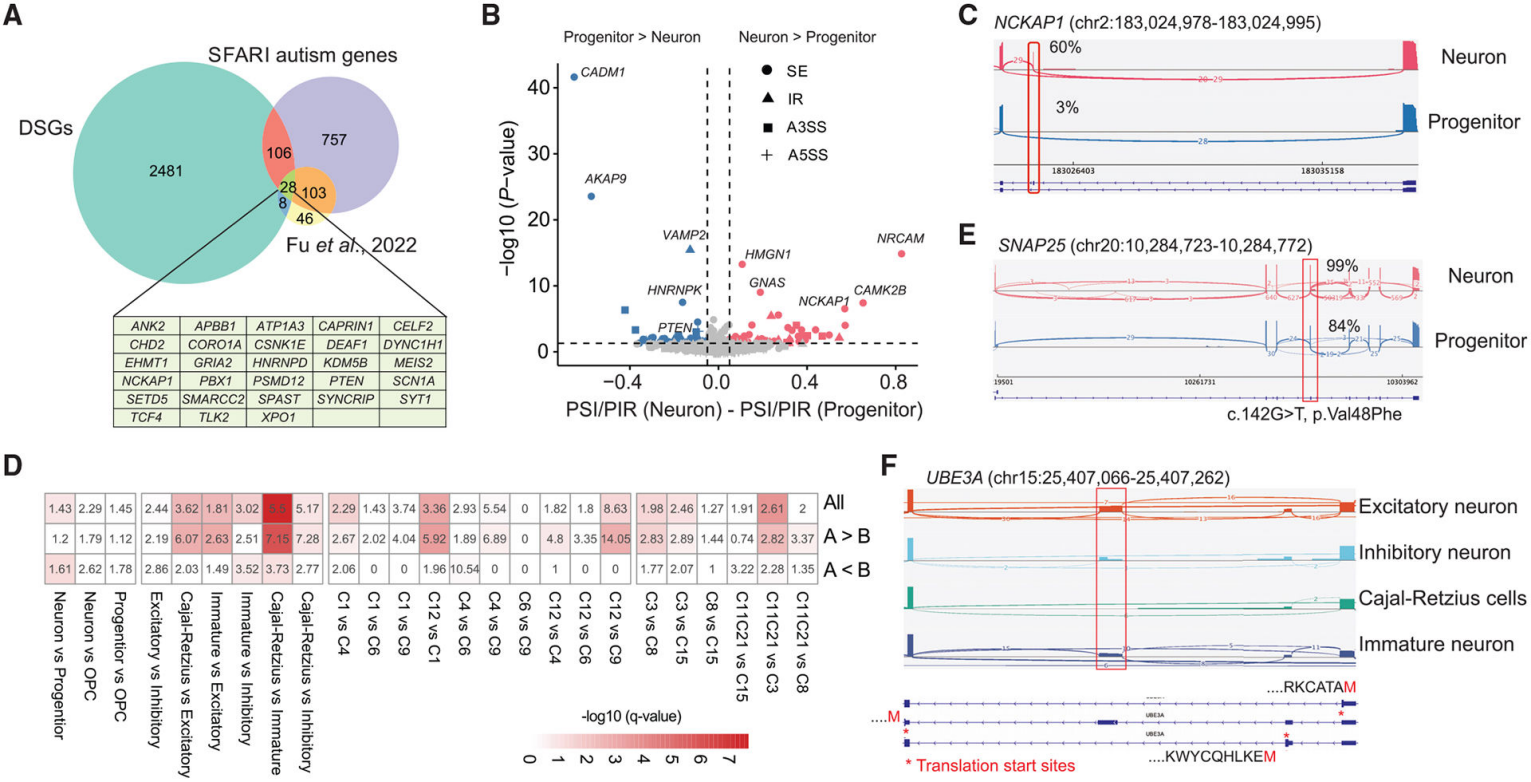
ASD genes are differentially spliced across cell types (A) Venn diagram showing genes with DSEs between cell types, ASD risk genes,^52^ and SFARI autism genes (994 genes, scores 1 and 2, and syndromic). DSGs, differentially spliced genes. (B) A volcano plot showing the DSEs in SFARI genes between neurons and progenitors. (C) A sashimi plot showing a DSE in the *NCKAP1* gene. (D) Enrichment analysis of DSEs for NMD-sensitive exons. A > B indicates that DSEs are more included in the first group being compared. For instance, in the comparison between neurons and progenitors, A > B indicates the DSEs that are more included in neurons, and vice versa. The odds ratios and p values were calculated based on Fisher’s exact tests. (E) A sashimi plot showing a DSE in the *SNAP25* gene, which contained a *de novo* missense mutation in an ASD proband.^54^ (F) An NMD-sensitive DSE in the UBE3A gene is predicted to introduce an alternative translation start site in neurons.

We further examined the splicing pattern of specific ASD genes cataloged in the SFARI database (evidence score of 1 or 2 or syndromic)^51^ or identified in a recent study (185 genes at FDR ≤ 0.05).^52^ We found that 142 ASD genes were differentially spliced between neural cell types (Figures 5A and 5B), such as *SPAST* and *PTEN* (Figure S5B). For example, a 45-bp exon (exon3) in *PTEN* showed higher inclusion in progenitors than in neurons (Figure S5B). Meanwhile, 209 co-regulated exon pairs contained at least one DSE, including nine pairs in four ASD-associated genes (*CELF2*, *FAM98C, HNRNPR,* and *PCM1*) (Table S5). We next examined PSD proteins, which constitute about a quarter of these 142 ASD genes (n = 33). For instance, *NCKAP1* regulates neuronal cytoskeletal dynamics, and disruptive variants in *NCKAP1* have been shown to cause ASD.^53^ We found an 18-bp microexon (exon 2) in *NCKAP1* that was included in neurons but skipped in progenitors (Figure 5C). The inclusion level of this microexon was lower in the neocortices of individuals with ASD compared to control individuals.^12^ These results identified cell-type-specific splice isoforms of ASD genes.

Inclusion or exclusion of DSEs can alter local amino acid sequences or induce nonsense-mediated mRNA decay (NMD) that turns gene expression up or down.^55^ We compared the 2,393 DSEs to previously identified NMD-sensitive exons^56^ and identified 155 NMD-sensitive DSEs, including alternative exons in *HNRNPDL, CLTA*, and *SRSF3* (Figure S5C). For example, the ultraconserved unproductive exon 4 in *SRSF3*^57^ was differentially spliced between neurons and progenitors and other cell types (Figure S5C). GO analysis of genes with NMD-sensitive DSEs suggested that RNA-splicing-related processes were highly enriched. Using the non-DSEs in each comparison as negative controls, DSEs between neurons and progenitors exhibited significant enrichment in the NMD-sensitive exons, and there were more NMD-sensitive exons in mature neurons than in immature neurons (Figure 5D). In addition, we identified five ASD genes (*SNAP25, HNRNPD, SF3B1, METTL26,* and *JMJD1C*) in which AS was potentially coupled with NMD. For example, we found a DSE (chr20:10,284,724–10,284,772) in the *SNAP25* gene that showed higher inclusion in neurons than in progenitors. The exclusion of the *SNAP25* DSE was predicted to introduce a frameshift and generate a premature stop codon in the downstream exon. Meanwhile, the DSE also contained a *de novo* missense mutation^54^ (c.142G>T [p.Val48Phe]; Figure 5E), which was predicted to be deleterious (Sorting Intolerant From Tolerant [SIFT] value = 0.01 and Polymorphism Phenotyping [PolyPhen] value = 0.997) and potentially affects the SNAP25 protein function. When relaxing the coverage threshold of DSEs to 8, we found that the *UBE3A* gene harbored a cryptic exon that was co-regulated with its upstream exon and introduced an alternative translation start site in excitatory neurons (Figure 5F). Furthermore, we found a DNM (chr16:635,776: A>C) in the acceptor site of the NMD-sensitive DSE (chr16:635,612–635,774) in *METTL26,* and the mutation was predicted to disrupt the splice acceptor (SpliceAI score = 0.85 and MMsplice delta_logit_psi score = −1.93) (Figure S5D). These observations suggest that cell-type-specific NMD exons regulate gene expression and may contribute to autism pathogenesis.

Increased IR downregulates gene expression and is considered a post-transcriptional signature associated with neurological diseases.^47,58^ We found 54 DRIs in ASD genes, such as *BAZ2B, CORO1A, ELAVL3,* and *VAMP2.* The 3′-terminal intron of *VAMP2* was partially included in progenitors but not in neurons (Figure S5E). This is consistent with a previous report that the homologous mouse *Vamp2* intron is retained.^59^

In summary, our scIso-seq analyses of human iPSC-derived cerebral organoids uncovered 31,181 previously unannotated isoforms and 4,820 unannotated exons, including 111 unannotated exons and 1,527 unannotated splice isoforms in ASD-associated genes. We identified 4,531 cell-type-specific events (2,393 SEs, 251 A3SSs, 461 A5SSs, and 1,426 IRs), and ASD-associated genes were highly enriched for splicing changes between neural progenitors and neurons. In total, 142 ASD genes displayed cell-type-specific isoforms. The results presented here describe transcript diversity in human neural development, highlight the regulation of ASD genes by cell-type-specific splicing, and provide an updated reference to interpret the functional impact of genetic variants identified in neurological disorders.

## DISCUSSION

AS has been recognized as a major mechanism in tissue-specific gene regulation.^4,60,61^ Single-cell/-nucleus studies such as the Human Cell Atlas uncovered unprecedented cell-type heterogeneity in human development,^15^ and single-nucleus Iso-seq has been reported to study transcript diversity in adult human brains.^62^ It remains unclear how AS is regulated between cell types during human neural development. Our scIso-seq analysis of cerebral organoids here uncovers over 31,000 uncatalogued isoforms and hundreds of cell-type-specific splicing events, significantly expanding the splicing landscape.

Dysregulation of AS networks has been associated with ASD pathogenesis. Our analysis uncovered 111 previously unannotated exons and 1,527 unannotated splice isoforms in ASD-associated genes. PTEN suppresses PI3Kinase signaling and is critical for brain development.^32^ *PTEN* mutations are causal for ASD, and we have identified 8 unannotated isoforms that were predicted to regulate critical protein domains (Figure 1F), suggesting that a fraction of the previously unannotated splice isoforms would critically regulate protein expression.

AS can turn on/off gene expression through NMD.^63,64^ We examined NMD-sensitive exons and found at least four ASD-associated genes were associated with AS-NMD. Copy number variations in the *UBE3A* gene are linked with ASD,^65^ and our results indicate that *UBE3A* is regulated by AS during neural development. The NMD exon in *UBE3A* showed higher inclusion in excitatory neurons than in other neuron subclasses. Suppressing AS-NMD exons has been shown to upregulate protein expression and alleviate haploinsufficiency.^66,67^ The *UBE3A* and other NMD exons identified here nominate potential targets for therapeutic interrogation by redirecting AS.

We analyzed the regulatory mechanism of cell-type-specific cassette exons during neurogenesis: sequence enrichment analysis identified binding motifs of master splicing regulators such as PTBP1 and RBFOX proteins around exons enriched in progenitors and neurons. Interestingly, we found that a potential HuR/ELAVL1-binding motif was highly enriched around neuron-enriched exons, while an A-rich motif was enriched downstream. These observations were consistent with higher HuR levels in dividing neural progenitors (Figure 2F) and suggest its role in suppressing neuronal exon inclusion.

It is challenging to study IR using short-read sequencing due to repetitive sequences.^44^ In principle, long-read sequencing covers the full transcript and provides a promising solution. Our analysis of human cerebral organoids uncovered over 1,400 differential IR events in oligoT-captured transcripts, suggesting the robustness. The results in this study significantly expanded transcript annotation and will provide a cell-type-specific full-transcript reference to study gene regulation in human neural development and disorders.

### Limitations of the study

While scIso-seq uncovered cell-type-specific splice forms in human cerebral organoids, limitations remain: (1) major cell types were reproducible between iPSC lines derived from different individuals, but variations in the cell-type proportions were frequently seen. This was caused by either genetic variations or technical biases introduced during iPSC production or organoid induction; how to minimize variations between organoids remains an actively studied area. (2) iPSC-derived human cerebral organoids are good models of human neural development, but the *in vitro* culture is different from the developing brain, such as its lack of vasculature and limited neuronal differentiation. (3) Internal priming of scRNA-seq oligonucleotides can introduce artifacts and partial transcripts. While multiple strategies such as CAGE and polyA signals have been employed to mitigate such effects, additional experimental and computational tools are required to address these limitations further.

## STAR★METHODS

### RESOURCE AVAILABILITY

#### Lead contact

Further information and requests for resources and reagents should be directed to and will be fulfilled by the lead contact, Xiaochang Zhang (xczhang@uchicago.edu).

#### Materials availability

This study did not generate any new unique reagents.

#### Data and code availability

- The sequencing data of scRNA-seq and PacBio Iso-Seq have been deposited to Sequence Read Archive (SRA) at the National Center for Biotechnology Information (NCBI) under accession number PRJNA947248.
- All original code supporting this study is available at https://github.com/yalanyang/scIso-seq_organoids.
- Any additional information required to reanalyze the data reported in this work paper is available from the lead contact upon request.

### EXPERIMENTAL MODEL AND STUDY PARTICIPANT DETAILS

#### Human iPSCs

Human iPSCs 19101 (from a male), 21792 (from a female), and 28126 (from a male) were shared by Yoav Gilad’s lab at the University of Chicago. These cell lines were characterized when they were first generated, including karyotyping, pluripotency, and the ability to differentiate into three germ layers.^75-77^ iPSCs were grown in Essential 8 (Thermo Fisher, A1517001) with penicillin-streptomycin (100U/mL, Thermo Fisher, 15140122) in 10-cm dishes coated with GelTrex (Thermo Fisher, A1413301) in an incubator with 5% CO_2_ at 37°C. Culture media were refreshed every day. iPSCs reaching about 90% confluence were dissociated with Accutase (Sigma-Aldrich, A6964) for passaging or the induction of cerebral organoids. A Rock inhibitor Y-27632 dihydrochloride (10 μM) (Tocris, 1254) was added to the culture media for the first 24 h after dissociation.

#### Human cerebral organoid

Brain organoids were induced based on a published protocol,^29^ with some modifications. Briefly, 3×10^6^ iPSCs were seeded per AggreWell 800 well (STEMCELL Technologies, 34815) in Essential 8 supplemented with Y-27632 dihydrochloride (10 μM) (Tocris, 1254) and penicillin-streptomycin (100 U/mL). 24 hour (h) later, iPSC spheroids were transferred into ultra-low attachment 6-well plates and incubated in Essential 6 supplemented with dorsomorphin (2.5 μM, Sigma-Aldrich, P5499) and SB-431542 (10 μM, Tocris, 1614) for 6 days to induce neural spheroids, which were then incubated in Neurobasal A medium (Thermo Fisher Scientific, 10888022) supplemented with B-27 minus vitamin A (1:50, Thermo Fisher Scientific, 12587010), GlutaMax (1:100, Thermo Fisher Scientific, 35050-061), epidermal growth factor (EGF, 20 ng/mL, R&D Systems, 236-EG), basic fibroblast growth factor (bFGF, 20 ng/mL, R&D Systems, 233-FB), and penicillin-streptomycin (100 U/mL) for 19 days. After that, the EGF and bFGF were replaced by brain-derived neurotrophic factor (BDNF, 20 ng/mL, PeproTech, 450-02) and NT-3 (20 ng/mL, PeproTech, 450-03) for 18 days. From the 43^rd^ day, brain organoids were incubated in Neurobasal A media supplemented with B-27 minus vitamin A (1:50), GlutaMax (1:100) and penicillin-streptomycin (100 U/mL) for long-term culture. Starting from the sixth day, plates containing spheroids or organoids were put on a shaker (40 rpm) in an incubator with 5% CO_2_ at 37°C.

### METHOD DETAILS

#### Characterization of cerebral organoids

To evaluate the growth of cerebral organoids, bright-field images were captured on a microscope (Invitrogen EVOSFL) at day 0, day 14, day 34, and day 60. The diameters of cerebral organoids were measured by ImageJ (https://imagej.net/ij/index.html). For immunostaining, cerebral organoids were fixed for 5 h in 4% paraformaldehyde (PFA) at 4°C. After fixation, cerebral organoids were rinsed with 1x Dulbecco’s phosphate buffered saline (DPBS) (Thermo Fisher Scientific, 14190-144) once for 15 minutes (min) at room temperature, cryopreserved in 30% (w/v) sucrose solution for 24 h at 4°C, and embedded in OCT (Sakura Finetek, 62550-01). 14 μm-thick sections of cerebral organoids were captured on glass slides using a cryostat (Thermo Fisher Scientific, NX50). After removing OCT/sucrose by 1x DPBS washing for 5 min, sections were incubated in blocking solution (1x DPBS containing 0.03% Triton X-100 and 5% normal donkey serum) at room temperature for 1 h, and further incubated with primary antibodies diluted in blocking solution overnight at 4°C. Primary antibodies targeting PAX6 (Abcam, ab5790, 1:100), NKX2-1 (Thermo Fisher, MA5-13961, 1:100), and SOX2 (Santa-Cruz, sc-17320,1:100) were used to label neural progenitors. Primary antibodies targeting RELN (R&D Systems, AF3820-SP, 1:100), TBR1 (Abcam, ab31940, 1:500), CTIP2 (Abcam, ab18465, 1:500), and SATB2 (Abcam, ab51502, 1:250) were used to label different types of neurons. After three times of 5-min washing with 1x DPBS, sections were incubated for 1 h at room temperature with fluorophore-conjugated secondary antibodies in the dark. Secondary antibodies were diluted at 1:500 in the blocking solution. Donkey anti-Rabbit Alexa Fluor 488 (Invitrogen, A-21206), Donkey anti-Rabbit Alexa Fluor 594 (Invitrogen, A-21207), Donkey anti-Mouse Alexa Fluor 488 (Invitrogen, A-21202), Donkey anti-Mouse Alexa Fluor 594 (Invitrogen, A-21203), Donkey anti-Rat Alexa Fluor 488 (Invitrogen, A-21208), Donkey anti-Goat Alexa Fluor 647 (Invitrogen, A-21447) were used for corresponding primary antibodies. After three times of 5-min washing with 1x DPBS, cerebral organoid sections were mounted with DAPI Fluoromount-G (SouthernBiotech, 0100-20). Cerebral organoid sections were scanned with a Leica SP8/Stellaris8 confocal microscope. Antibodies used in this study were listed in the key resources table.

#### 10x genomics scRNA-seq and PacBio Iso-Seq library preparation

The disassociated cells were captured on the 10x Genomics Chromium controller. Sequencing library construction was performed using the Chromium Single Cell 3′ Library and Gel Bead Kit v3 and sequenced on an Illumina NovaSeq 6000 with PE 2 × 50 paired-end reaction. In parallel, the untagmented full-length cDNA (3-min PCR extension instead of 1 min) was used for long-read sequencing with PacBio Sequel II. PacBio library preparation was performed with 1 μg amplified cDNA using SMRTbell Express Template Prep Kit V2.0. We sequenced two PacBio Sequel II 8M SMRT cells for 19101 and 28126 and three cells for 21792.

### QUANTIFICATION AND STATISTICAL ANALYSIS

#### scRNA-seq pre-processing, initial analysis and clustering

For each organoid, the sequenced libraries were processed according to Cell Ranger (version 6.1.2) *count* and *aggr* (aggregation) pipelines. We used the Seurat package (v4.2.0)^70^ in R (v4.1.2) and filtered out cells with detected genes over 5,000 or below 300 or that had over 5% mitochondrial gene counts. The filtered matrix was normalized using Seurat’s *LogNormalize* function with default parameters and the top 2,000 variable genes were identified using the *FindVariableFeatures* ‘vst’ method. After pre-processing, cells from the four organoids were integrated using the *FindIntegrationAnchors* and *IntegrateData* functions to obtain a combined and centered expression matrix. Principal component analysis was carried out on this centered expression matrix. The top 30 principal components were used to build a shared nearest neighbor (SNN) graph, which was then clustered using the Louvain algorithm (resolution = 0.7) implemented in the Seurat *FindClusters* function. We applied the UMAP algorithm with the number of neighbors set to 30 to visualize the raw data following the Seurat3 default implementation. We identified marker genes for each cluster using the Seurat *FindMarkers* function with default parameters. The top marker genes were used to assign cell type annotations for each cell cluster.

#### Iso-Seq data analysis

The raw long-read fastq sequences were processed using the Iso-seq3 (v3.8.0) pipeline (https://github.com/PacificBiosciences/IsoSeq). Briefly, we used Isoseq3 ccs module to generate circular consensus sequence (CCS) reads from the sub-reads generated from the sequencing run. 5′ and 3’ cDNA primers were removed using Lima (v2.6.0) to generate FL reads, followed by extraction of the UMIs and single-cell cell barcodes using Isoseq3 tag. Then the polyA tail and concatemers from FL read were removed by Isoseq3 refine to generate FLNC transcripts. PCR artifacts were deduplicated to generate high-quality transcripts using isoseq3 groupdedup with the parameters “–max-tag-mismatches 1 –max-tag-shift 0”. High-quality, full-length transcripts were then mapped to the human genome (hg38) using minimap2 (version 2.17-r974-dirty) with the following parameters: -ax splice -t 30 -uf –secondary = no^71^ and collapsed to unique isoforms using the cDNA Cupcake Package. After filtering away isoforms supported by fewer than two FLNC reads, further quality control, isoform annotations and artifacts removal were performed using SQANTI3 (v5.1.1).^72^ Several characteristics provided by SQANTI3 were used to assess the reliability of the full-length isoforms, including predicted reverse transcriptase template switching (RTS) artifacts, canonical splice site, overlap of 3′ ends with poly(A) tails, and 5′ transcript ends with independently published Cap Analysis of Gene Expression (CAGE) data. The poly(A) sites and CAGE peaks were downloaded from PolyASite 2.0^68^ and refTSS,^69^ respectively. Protein domains of the full-length isoforms were predicted with SMART.^78^ To validate the unannotated exons and isoforms identified in the Iso-Seq, we conducted a comparison with the annotation gtf file derived from previously published long-read data of the human cerebral cortex^33^ using gffcompare.^79^ The isoforms and exons with "class_code " = "" were considered to be validated.

#### Exon coordination analysis

Alternative exons that met the following criteria were used for exon coordination analysis: (1) 0.05 < PSI <0.95 in the Iso-Seq; (2) ≥20 supporting FL reads (inclusion + exclusion); (3) the first and the last exons of each gene were excluded. For all alternative exon pairs within a gene, we generated a matrix which counted the number of reads for both exons included (in–in), only the first exon included (in–out), only the second exon included (out–in), and both exons excluded (out–out). Using this matrix, we tested the co-association of each exon pair by fisher’s exact test and adjusted the p values by Benjamini–Yekutieli method. The odds ratio (OR) was calculated using the following formula: OR = [(in–in+1) × (out–out+1)]/[(in–out+1) × (out–in+1)].

#### Differentially spliced events between cell types

Single-cell barcodes in long reads were used to determine the cell origin. Given that most single cells were assigned to a specific cell cluster, we were also able to assign the cell cluster/type information to each long read. We used FLAIR (v2.0)^34^ to quantify transcripts and identify differential AS events between cell types including skipped exon (SE), alternative 5′ splice site (A5SS), and alternative 3′ splice site (A3SS). A “percentage spliced in” (PSI) value was assigned to each exon or splice site by estimating its abundance compared to adjacent exons. Significant differential splicing events were defined as ≥ 10 supporting FL reads (inclusion + exclusion), ∣ΔPSI∣ ≥ 5% and adjusted *p* value <0.05. The percent intron retention (PIR) was calculated by dividing intron-retained isoform abundance by total transcript abundance. Differentially retained introns between cell types were identified with IRFinder-S (v2.0.0)^45^ using cutoffs of ∣ΔPIR∣ ≥ 5% and adjusted *p* value <0.05. The raw RNA-seq data of mouse excitatory neurons and interneuron subtypes were downloaded from NCBI GEO (GSE122100),^46^ differentially retained introns between excitatory neurons and interneuron subtypes were identified using DESeq2 algorithm in IRFinder-S with cutoffs of ∣log2 (Fold Change)∣ ≥ 1 and adjusted *p* value <0.05.

#### Motif analysis

We performed motif discovery and enrichment analysis with the 300-bp downstream and upstream introns of differentially skipped exons using default parameters of XSTREME.^36^ For each region, we used exons with higher PSI in neurons as foreground, and exons with higher PSI in progenitors as background, and vice versa.

#### Disease-associated genes and exons

The known ASD-associated gene lists were selected from the SFARI Autism database (https://gene.sfari.org/, 05-05-2022release).^51^ Genes with evidence scores of 1, 2 or syndromic were used in this study. Disease-associated exons were extracted from previous studies: 1,776 skipped exons from a comparison of ASD cases with controls (p < 0.05),^12^ 506 ALS-associated skipped exons by comparing C9orf72 ALS brains with control brains,^47^ and 1,107 Schizophrenia-associated skipped exons.^48^ Coordinates of regions were converted between hg19 and hg38 using the LiftOver tool from UCSC.

#### Cell type and literature curated gene set enrichment analyses

For gene set enrichment studies, fisher’s exact tests were performed to calculate *p* values on gene lists against curated gene lists: RBFOX1 target genes,^37^ FMRP target genes,^38^ HUR target genes,^39^ the genes encoding proteins of the postsynaptic density (PSD),^35^ and NMD-sensitive exons.^56^ Gene Ontology (GO) enrichment were analyzed with DAVID v6.8.^80^

#### Properties of retained introns

The GC content of introns was calculated using the nuc option in BEDTools.^81^ The strength of 5′ splice sites and 3′ splice sites were calculated using MaxEntScan with the maximum entropy score and default parameters.^82^ PhastCons scores based on the multiple alignment of 100 vertebrate species were obtained from the UCSC genome browser. For each retained intron, the bigWigAverageOverBed function from the UCSC Utilities package calculates the average conservation score across all base pairs.

#### *De novo* mutations around DSEs

To assess the potential effect of these variants on splicing, we extracted human *de novo* variants in ASD from denovo-db (v.1.6.1)^83^ and intersected the coordinates of *de novo* variants with the 300-bp upstream and downstream introns of DSEs. The effects of *de novo* variants on splicing were evaluated using two tools, SpliceAI^73^ and MMsplice.^74^ To analyze the enrichment of *de novo* variants in and around DSEs, the *de novo* mutations in ASD probands and siblings were extracted from a recent whole genome sequencing of 42,607 autism cases.^84^

### ADDITIONAL RESOURCES

The scIso-seq data of human cerebral organoids is publicly available via a web interface (https://zlab1.shinyapps.io/scIsoseq/) that allows for search and visualization of the data.

## Supplementary Material

1

2

3

4

5

6

## Figures and Tables

**Table:** KEY RESOURCES TABLE

REAGENT or RESOURCE	SOURCE	IDENTIFIER
Antibodies		
Anti-PAX6	Abcam	Cat# ab5790; RRID: AB_305110
Anti-NKX2-1	Thermo-Fisher	Cat# MA5-13961; RRID: AB_10984070
Anti-Ki67	Abcam	Cat# ab15580; RRID: AB_443209
Anti-SOX2	Santa Cruz Biotechnology	Cat# sc-17320; RRID: AB_2286684
Anti-TBR1	Abcam	Cat# ab31940; RRID: AB_2200219
Anti-CTIP2	Abcam	Cat# ab18465; RRID: AB_2064130
Anti-RELN	R&D Systems	Cat# AF3820; RRID: AB_2253745
Anti-SATB2	Abcam	Cat# ab51502; RRID: AB_882455
Donkey anti-Rabbit IgG (H + L) Highly Cross-Adsorbed Secondary Antibody, Alexa Fluor^™^ 488	Invitrogen	Cat# A-21206; RRID: AB_2535792
Donkey anti-Rabbit IgG (H + L) Highly Cross-Adsorbed Secondary Antibody, Alexa Fluor^™^ 594	Invitrogen	Cat# A-21207; RRID: AB_141637
Donkey anti-Mouse IgG (H + L) Highly Cross-Adsorbed Secondary Antibody, Alexa Fluor^™^ 488	Invitrogen	Cat# A-21202; RRID: AB_141607
Donkey anti-Mouse IgG (H + L) Highly Cross-Adsorbed Secondary Antibody, Alexa Fluor^™^ 594	Invitrogen	Cat# A-21203; RRID: AB_141633
Donkey anti-Rat IgG (H + L) Highly Cross-Adsorbed Secondary Antibody, Alexa Fluor^™^ 488	Invitrogen	Cat# A-21208; RRID: AB_2535794
Donkey anti-Goat IgG (H + L) Cross-Adsorbed Secondary Antibody, Alexa Fluor^™^ 647	Invitrogen	Cat# A-21447; RRID: AB_2535864
Deposited data		
scRNA-seq and Iso-Seq of cerebral organoids	This study	SRA PRJNA947248
Iso-Seq of human cortex	Leung et al.,^33^	SRA: PRJNA664117
RNA-seq of mouse interneurons and excitatory neurons	Huntley et al.,^46^	GEO: GSE122100
RBFOX1 target genes	Weyn-Vanhentenryck et al.,^37^	http://doi.org/10.1016/j.celrep.2014.02.005
FMRP target genes	Darnell et al.,^38^	http://doi.org/10.1016/j.cell.2011.06.013
HUR target genes	Mukherjee et al.,^39^	http://doi.org/10.1016/j.molcel.2011.06.007
PSD genes	Iossifov et al.,^35^	http://doi.org/10.1038/nature13908
NMD-sensitive exons	Karousis et al.,^56^	http://doi.org/10.1186/s13059-021-02439-3
SFARI autism genes	SFARI	https://gene.sfari.org
Neurodevelopmental disorder genes	OMIM database	https://www.omim.org
ALS-associated skipped exons	Wang et al.,^47^	https://doi.org/10.1101/gr.265298.120
Schizophrenia-associated skipped exons	Takata et al.,^48^	https://doi.org/10.1038/ncomms14519
ASD-associated skipped exons	Parikshak et al.,^12^	http://doi.org/10.1038/nature20612
*De novo* variants	Zhou et al.,^50^	https://doi.org/10.1038/s41588-019-0420-0
poly(A) sites	Herrmann et al.,^68^	https://www.polyasite.unibas.ch
CAGE peaks	Abugessaisa et al.,^69^	https://reftss.riken.jp/reftss/Main_Page
Human reference genome Release 38 (hg38)	GENCODE	https://www.gencodegenes.org/human/release_40.html
Experimental models: Cell lines		
19101	Yoav Gilad lab, The University of Chicago	N/A
28126	Yoav Gilad lab, The University of Chicago	N/A
21792	Yoav Gilad lab, The University of Chicago	N/A
Software and algorithms		
Cell Ranger (version 6.1.2)	10x Genomics	https://support.10xgenomics.com/single-cell-gene-expression/software/downloads/latest
Seurat package (v4.2.0)	Hao et al.,^70^	http://doi.org/10.1016/j.cell.2021.04.048
R (v4.1.2)	The R Foundation	https://cran.r-project.org/
Iso-seq3 (v3.8.0)	Pacific Biosciences	https://github.com/PacificBiosciences/IsoSeq
Lima (v2.6.0)	Pacific Biosciences	https://lima.how
Cupcake	Pacific Biosciences	https://github.com/Magdoll/cDNA_Cupcake
Iso-seq3	Pacific Biosciences	https://github.com/PacificBiosciences/IsoSeq
minimap2 (version 2.17-r974-dirty)	Li,^71^	https://doi.org/10.1093/bioinformatics/btab705
SQANTI3 (v5.1.1)	Tardaguila et al.,^72^	http://doi.org/10.1101/gr.222976.117
FLAIR (v1.5.1)	Tang et al.,^34^	http://doi.org/10.1038/s41467-020-15171-6
IRFinder-S (v2.0.0)	Lorenzi et al.,^45^	http://doi.org/10.1186/s13059-021-02515-8
DAVID v6.8	DAVID Bioinformatics Resources	https://david.ncifcrf.gov
SpliceAI	Jaganathan et al.,^73^	http://doi.org/10.1016/j.cell.2018.12.015
MMsplice	Chen et al.,^74^	http://doi.org/10.1186/s13059-019-1653-z
Other		
Resource Website for publication	This paper	https://zlab1.shinyapps.io/scIsoseq/
R and Shell codes	This paper	https://github.com/yalanyang/scIso-seq_organoids

## Highlights

- Over 31,000 uncatalogued isoforms are identified in human cerebral organoids
- 4,531 cell-type-specific splicing events are identified in human cerebral organoids
- Alternative exons are enriched for autism-associated exons and *de novo* mutations
- 142 autism genes display cell-type-specific isoforms
