# Supplementary material for: Single-cell long-read sequencing in human cerebral organoids uncovers cell-type-specific and autism-associated exons
Source: Cell Rep. Author manuscript; Available in PMC 2024 Feb 5. (PMC10842930; doi:10.1016/j.celrep.2023.113335)
Supplement: 1 [file NIHMS1948188-supplement-1.pdf]

Cell Reports, Volume *42*

Supplemental information

Single-cell long-read sequencing in human cerebral organoids uncovers cell-type-specific and autism-associated exons

Yalan Yang, Runwei Yang, Bowei Kang, Sheng Qian, Xin He, and Xiaochang Zhang

Supplementary Figures

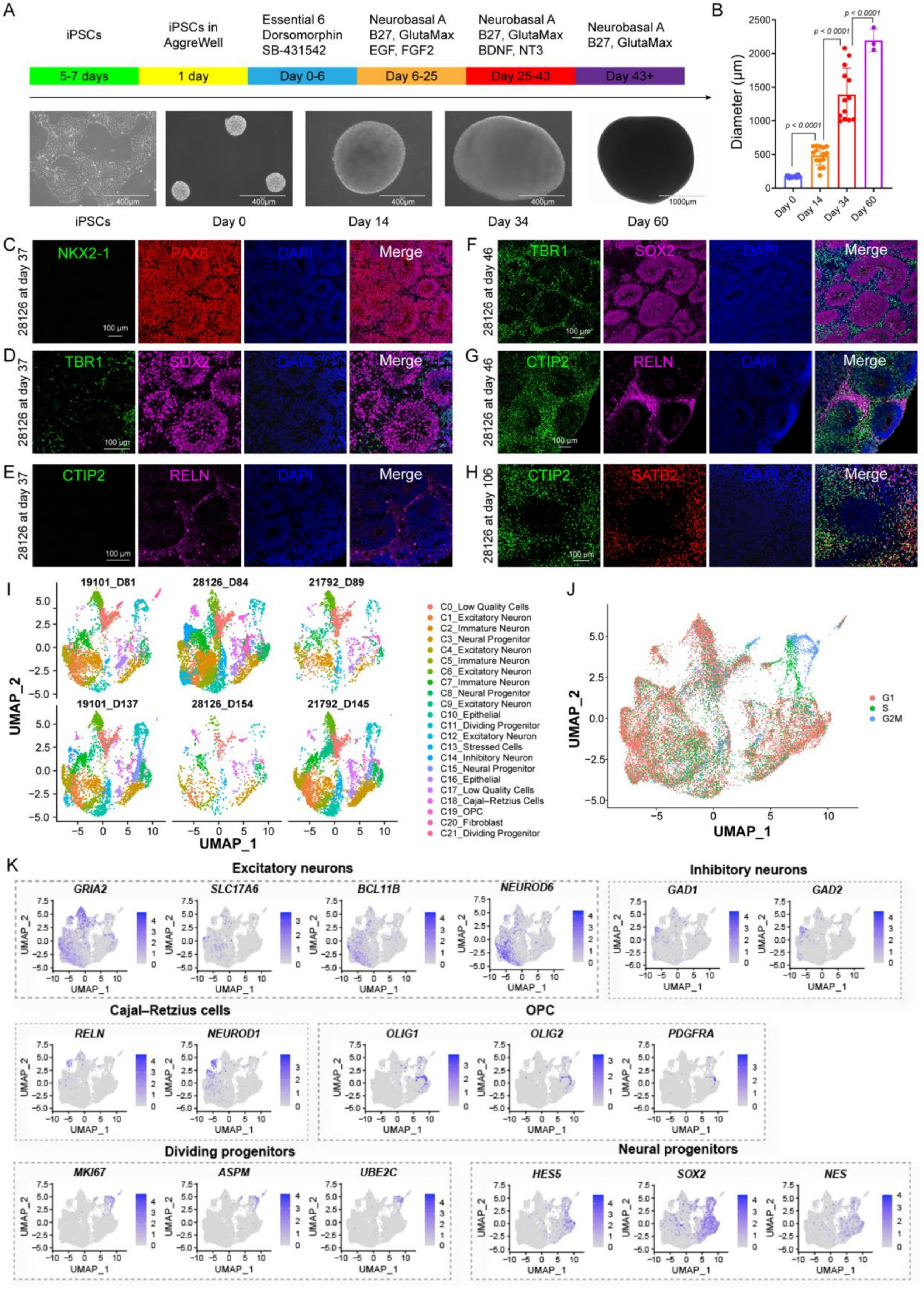

Figure S1. Generation and scRNA-seq analysis of cerebral organoids.

A. Workflow of cerebral organoids generation. This protocol began with the culture of iPSCs and then used SMAD pathway inhibitors dorsomorphin and SB-431542 to induce the neuralization of three-dimensional spheroids. Grow factors EGF and FGF2 were used to accelerate proliferation, and then BDNF and NT3 were used to promote cerebral organoid maturation. After 43-day differentiation, cerebral organoids were maintained in neurobasal A media supplemented with GlutaMax and B27.

B. Diameters of cerebral organoids at different stages. An ordinary one-way ANOVA test was performed between each group. N = 22, 15, 13, and 3 for day 0, day 14, day 34, and day 60, respectively.

C-H. Representative immunostaining images of cerebral organoids at different stages showing neural progenitors (PAX6 and SOX2) and neuron types (RELN, TBR1, CTIP2, and SATB2). Scale bar = 100 μm.

I. UMAP visualization of scRNA-seq data from each of the six batches of cerebral organoids. Cells are color-coded by annotated cell types. 19101_D137 (Day 137), 28126_D84, and 21792_D145 were subjected to Iso-Seq.

J. A feature plot showing the cell cycle status of each cell.

K. Feature plots showing the expression of marker genes of indicated cell types.

Related to Figure 1.

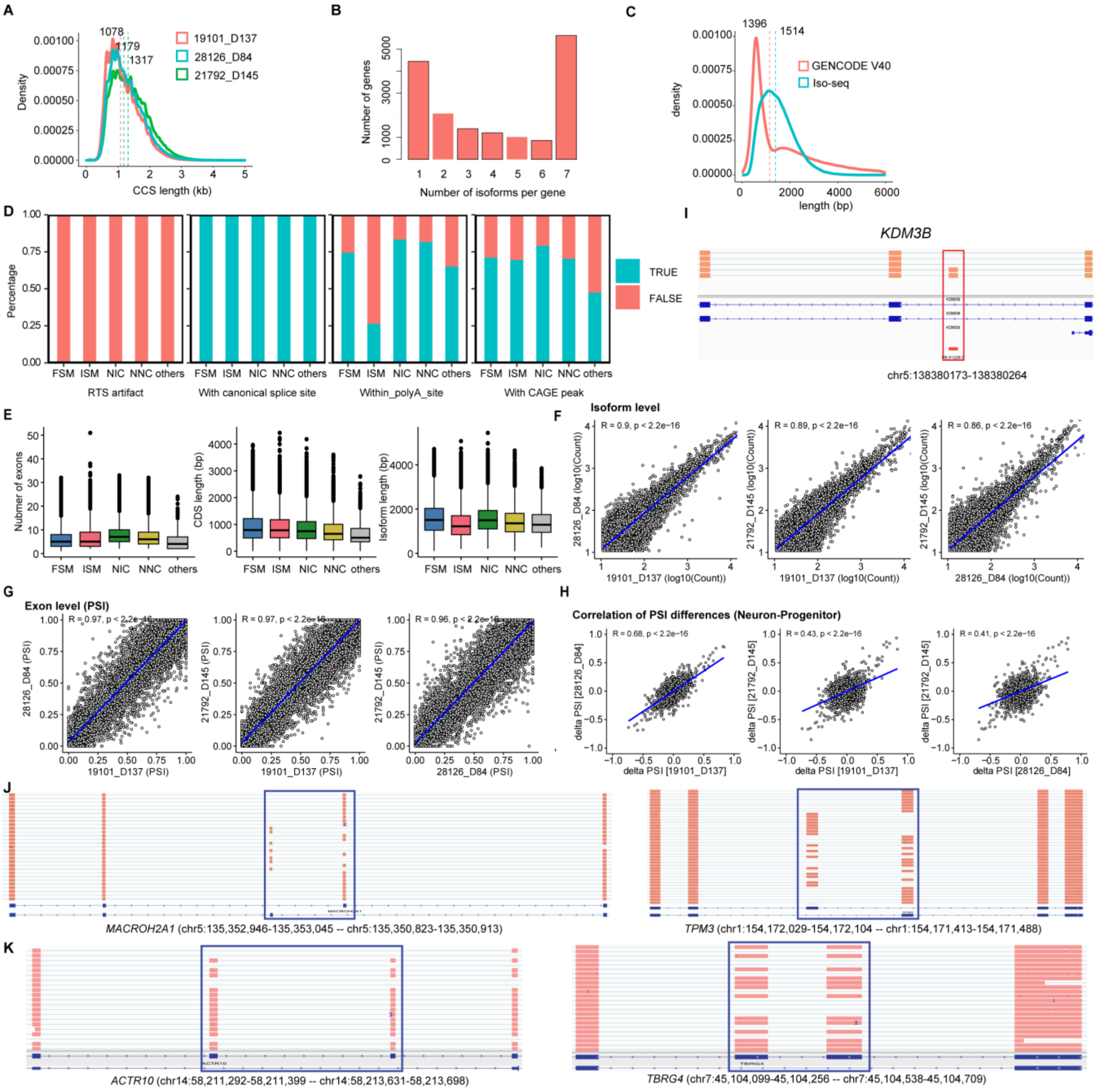

Figure S2. PacBio Iso-Seq captures full-length transcripts, unannotated splice isoforms, and coordinated splicing events.

A. The length distributions of CCS read generated by Iso-Seq.
B. A bar plot showing the number of isoforms per gene.
C. The length distributions of isoforms identified by Iso-Seq and those in GENCODE v40 annotation file (hg38). The median length of protein-coding isoforms in Iso-Seq and GENCODE is 1,514 and 1,396 bp, respectively.
D. Quality control metric showing the percentage of predicted full-length isoforms with reverse transcriptase template switching (RTS) artifacts, canonical splice sites, poly(A) sites, and CAGE peaks.
E. Characteristics of identified transcripts including the number of exons per transcript, the length of the coding sequence (CDS), and the length of individual isoforms.
F. The correlation between organoid samples at the isoform level based on read counts.
G. The correlation between organoid samples at the exon level based on PSI values.

H. The correlation of ΔPSI (Neuron - Progenitor) of alternative exons across organoid samples. The correlations were calculated by Pearson's correlation coefficient.
I. IGV browser tracks showing a previously unannotated exon identified in the *KDM3B* gene.
J. Mutually exclusive exons in *MACROH2A1* and *TPM3*.
K. Mutually inclusive exons in *ACTR10* and *TBRG4*.

Related to Figure 1.

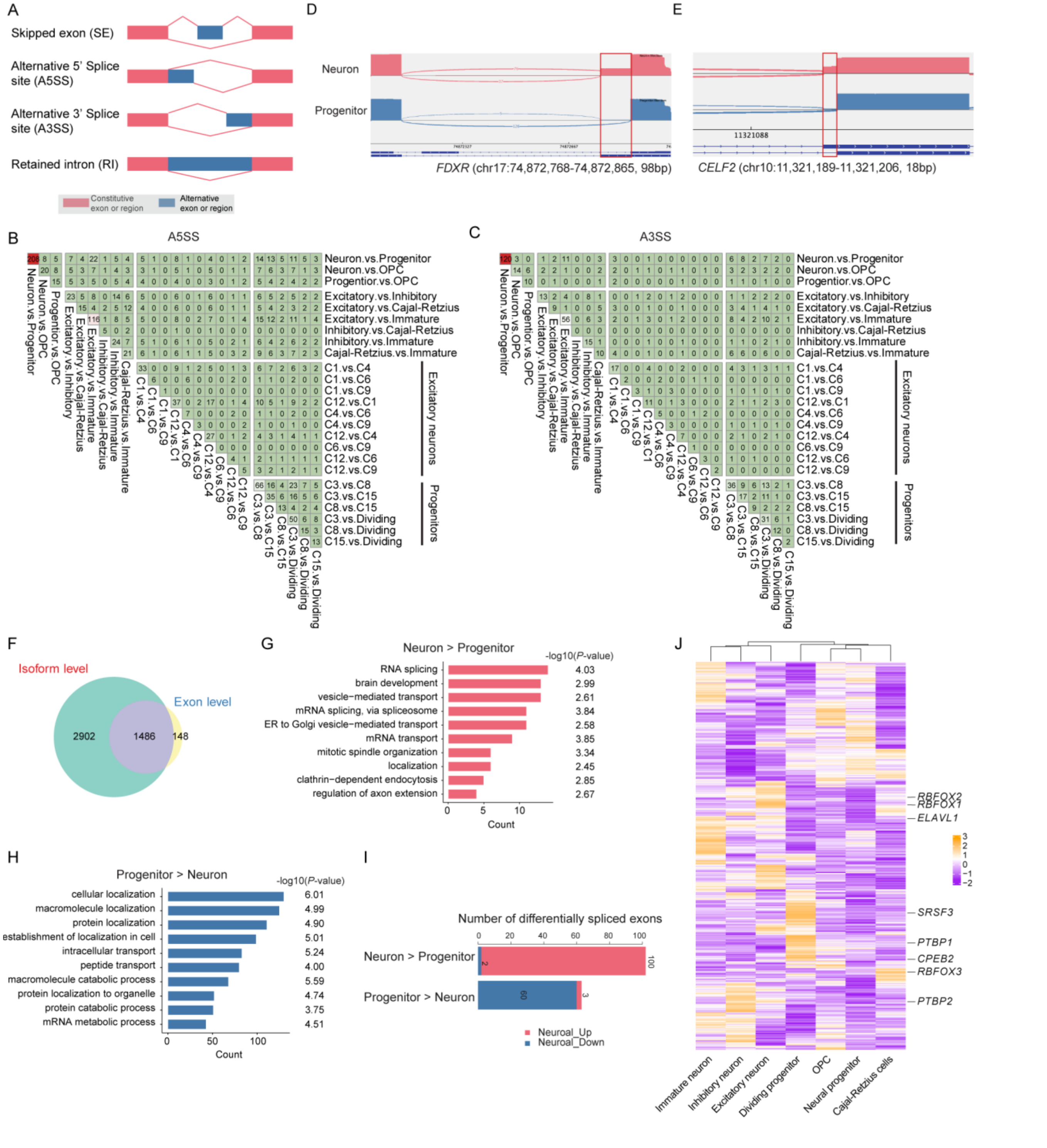

Figure S3. Identification of cell-type-specific A5SS, A3SS and SE events.

A. The schematic diagram of four types of splicing events.
B. Pairwise comparison showing differential A5SS events between different cell types.
C. Pairwise comparison showing differential A3SS events between different cell types.
D. Sashimi plots from scIso-Seq showing a differential A5SS in the *FDXR* gene.
E. Sashimi plots from scIso-Seq showing a differential A3SS in the *CELF2* gene.
F. Venn diagram showing the overlap of genes identified by significant differential isoform expression (isoform level test) with genes carrying significant DSEs (exon level test).
G. GO enrichment of genes with DSEs showing higher inclusion in neurons than in progenitors.

H. Enriched GO enrichment of progenitor-specific DSEs.
I. Comparison of DSEs identified in this study with those identified in a previous bulk RNA-seq study (PMID: 25525873).
J. A heatmap map showing differentially expressed RBPs across cell types in cerebral organoids. Each row indicates a single gene.

Related to Figure 2.

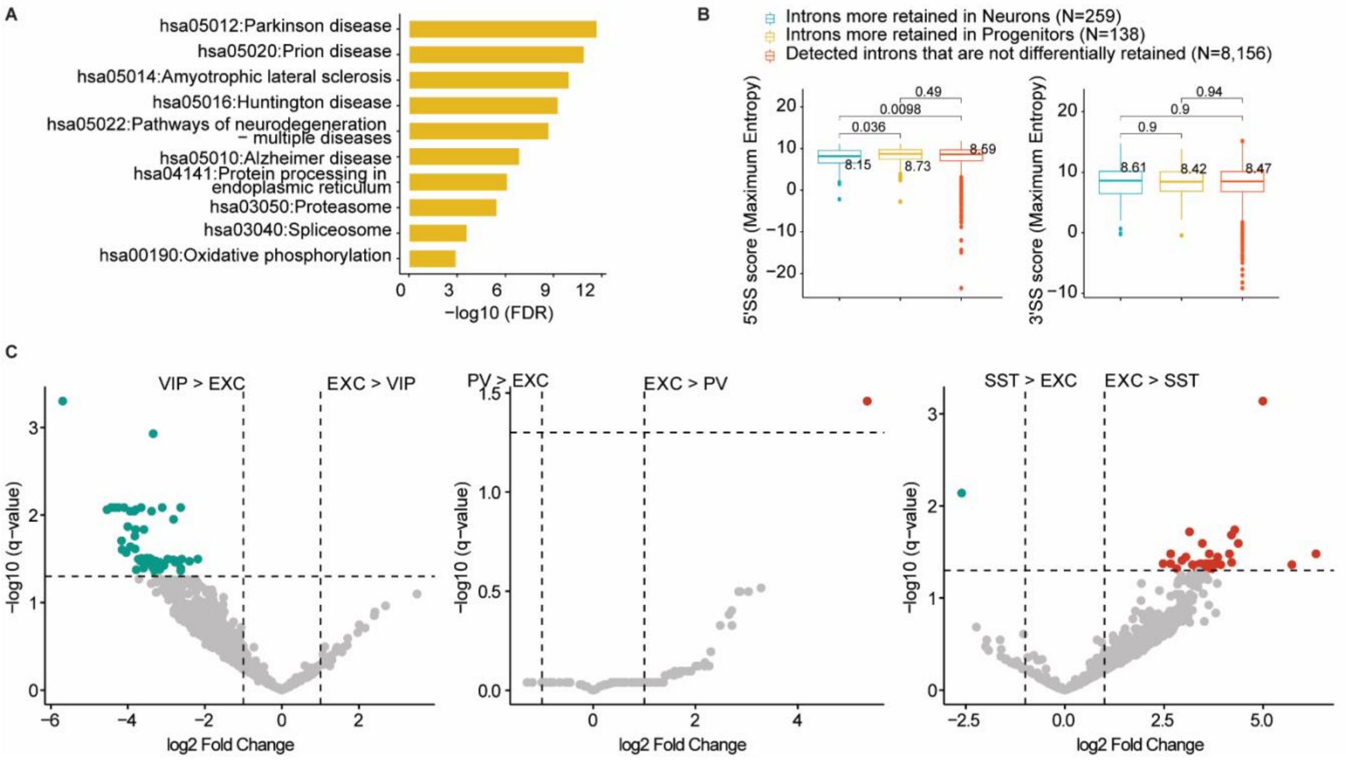

Figure S4. Differentially retained introns have weaker 5′ splice sites.

A. KEGG analysis of the genes with differentially retained introns.
B. Characteristics of differentially retained introns. Box plots showing the strength of the 5′ splice sites (5′ SS) and 3′ splice sites (3′SS) (as entropy scores) of introns that are more retained in neurons (cyan), progenitors (yellow), or not differentially spliced between neurons and progenitors (red). Mann-Whitney *U* test was used for statistical analysis.
C. Volcano plots showing the differentially retained introns between mouse interneurons and excitatory neurons based on re-analyses of a published RNA-seq dataset (GSE122100). The original report contained one type of excitatory neurons (EXC) and three types of interneurons: vasoactive intestinal peptide (VIP), parvalbumin (PV) and somatostatin (SST). Differentially retained introns between interneurons and excitatory neurons were identified by IRFinder-S using cutoffs of |log2 (Fold Change) | ≥1 and adjusted *P* value < 0.05.

Related to Figure 3.

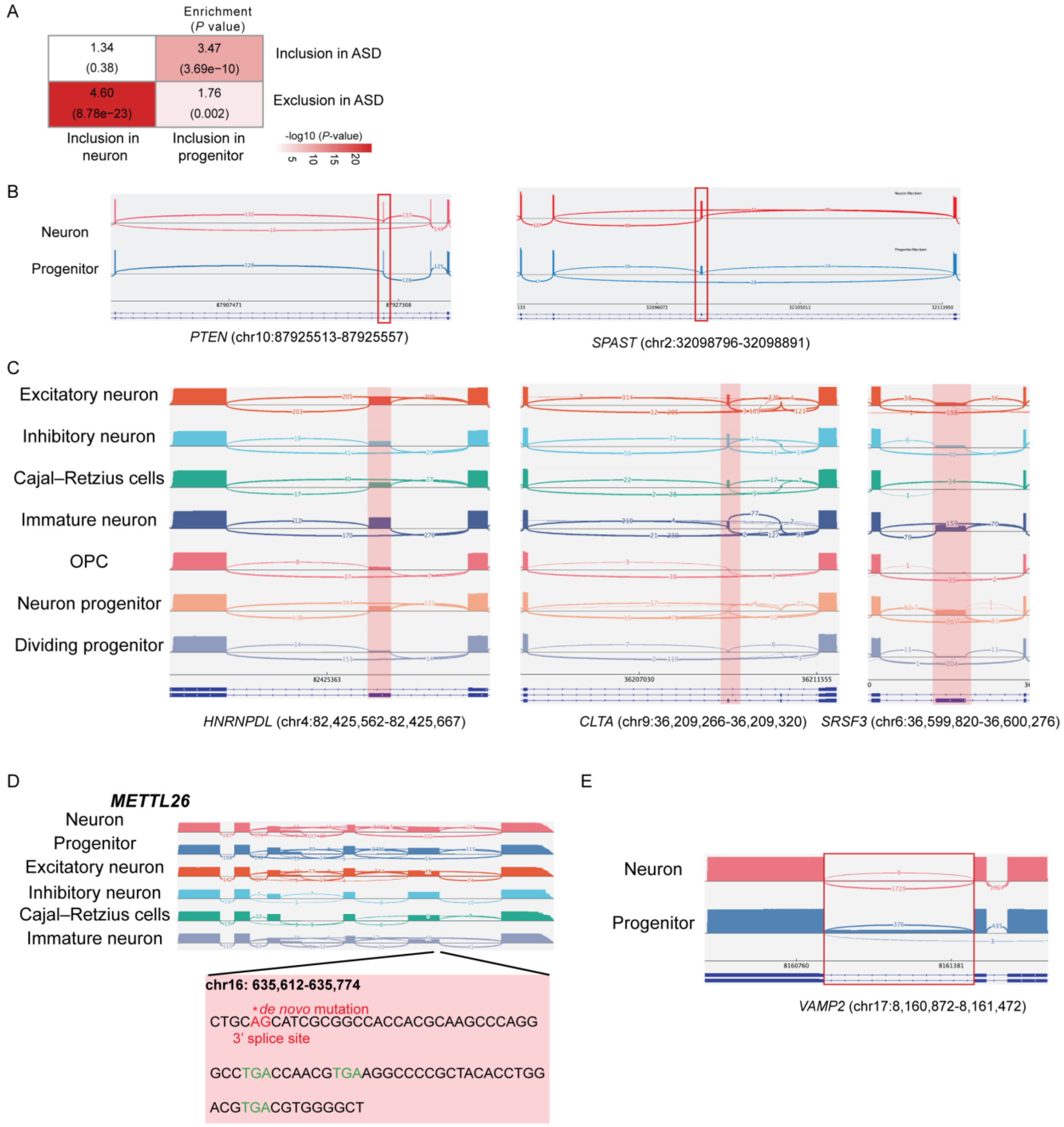

Figure S5. Cell-type-specific and NMD-sensitive exons, and *de novo* mutations in ASD genes.

A. Enrichment analysis of DSEs between neurons and progenitors with alternative exons identified in postmortem ASD brains. The odd ratios and *P* values were calculated based on Fisher's exact tests.
B. Sashimi plots from long-read Iso-Seq showing the alternatively spliced exons between neurons and progenitors in autism genes *SPAST* and *PTEN*.
C. Sashimi plots from long-read Iso-Seq showing NMD-sensitive DSEs in *HNRNPDL*, *CLTA*, and *SRSF3*.
D. A *de novo* mutation in *METTL26* disrupts the splice acceptor site of the NMD-sensitive DSE.
E. Sashimi plots from long-read Iso-Seq showing the differentially retained intron in the *VAMP2* gene.

Related to Figure 4 and Figure 5.
